# Exercise and cancer: return to work as a firefighter with ostomy after rectal carcinoma – a case report

**DOI:** 10.1097/MD.0000000000004309

**Published:** 2016-07-22

**Authors:** Joachim Wiskemann, Kai Schommer, Dirk Jaeger, Friederike Scharhag-Rosenberger

**Affiliations:** a Department of Medical Oncology, National Center for Tumor Diseases (NCT) Heidelberg; b Department of Sports Medicine, Heidelberg University Hospital, Heidelberg, Germany.

**Keywords:** colorectal cancer, endurance training, rehabilitation, stoma, strength training

## Abstract

**Background:**

Colorectal cancer survivors are deconditioned through anticancer therapy. Furthermore, about 10% of them have a permanent ostomy which is associated with weakened abdominal muscles and an increased risk of a hernia. This case study reports on how a firefighter with rectal carcinoma and ostomy was trained to regain operational fitness.

**Methods:**

A 44-year-old firefighter (178 cm, 82 kg) with an adenocarcinoma of the rectum (diagnosed 24 months prior) had been treated with neoadjuvant radiochemotherapy and surgery. After 2 temporary ileostomies, a permanent colostomy was performed 14 weeks before the start of a 9-months training program. The program included sensorimotor, endurance, and strength training of increasing volume and intensity. Endurance, strength, and patient reported outcomes were assessed every 2 to 3 months.

**Results:**

Training frequency varied from 1 to 3 sessions/week, although 3 to 5 sessions/week were prescribed. Peak power output was 150, 158, 167, 192, and 175 watts at baseline, 2, 4, 6, and 9 months. Maximal oxygen uptake increased from 1.56 L/min (19.0 mL/min/kg) to 2.39 L/min (28.8 mL/min/kg) after 6 months. Maximal isokinetic peak torque (MIPT) of the knee extensors were 138.0 and 196.5 Nm (Newton meter) at baseline and 6 months. MIPT of the elbow and hip flexors increased from 51.8 to 66.0 Nm and 213.8 to 239.7 Nm, respectively, after 6 months. Physical fatigue decreased by 65% and distress by about 50% after 9 months. The firefighter passed a test for occupational fitness after 6 months and was permitted to work with an exterior crew on a pump truck.

**Conclusion:**

It is possible for colorectal cancer survivors with ostomy to regain occupational fitness for physically demanding tasks like firefighting through an individually tailored and supervised training program.

## 1 Introduction

Cancer survivors with physically demanding occupations are often unable to work because physical fitness is severely reduced through the disease, side effects of the anticancer therapies, and through physical inactivity.^[1,2]^ In colorectal cancer (CRC) survivors, for example, abdominal stability is reduced after laparotomy and endurance capacity as well as strength is affected by chemotherapy and radiation.^[2]^ Beyond that, 1 year after surgery, temporary ostomy is quite frequent in CRC survivors with a rate of up to 35%^[3,4]^ or 50% (own unpublished data). A permanent ostomy is present in about 10% of CRC survivors. This is associated with permanently reduced abdominal stability and an increased risk of a hernia.^[3–5]^ Furthermore, it is related to various psychosocial problems (e.g., feeling depressed and tired, dissatisfaction with appearance, change in clothing, and worry about noises from the ostomy) and it negatively influences quality of life (QoL).^[3]^ Therefore, still being able to work in physically demanding professions appears desirable for CRC survivors with ostomy from both a psychosocial and an economic point of view. However, little is known about the trainability of this population.

Knowledge in the field of exercise and CRC comes from observational studies and randomized controlled trials (RCTs) investigating the effects of physical activity on health-related outcomes. Five observational studies demonstrate a relative risk reduction of up to 61% for cancer-related mortality and up to 57% for total mortality in the most physically active compared to the most physically inactive CRC patients.^[6–9]^ Interestingly, the effects of physical activity were not influenced by clinically relevant factors (age, gender, number of positive lymph nodes, body mass index, and chemotherapy agents) but were predominantly detected in patients with tumors at stages II and III. Despite these promising epidemiological data, only 6 randomized controlled exercise intervention trials have so far been conducted in this patient group.^[10,11]^ Their findings include increases in physical fitness and a reduced length of hospital stay. One reason for the small number of studies might be that there is a huge uncertainty with regard to exercisability of CRC patients having an ostomy. In the mentioned studies, information on the prevalence of ostomy is widely missing. A total of 3 RCTs reported data on this topic with 2 of them also including patients with other types of cancer. The colostomy rates were 9.7% (n = 9),^[12]^ 3.3% (n = 4),^[13]^ and 3.0% (n = 8)^[14]^ but no specific safety, feasibility, or adherence data with regard to exercise for this subgroup were reported.

Given the lack of knowledge with regard to professional rather than health-oriented exercise training in CRC survivors and the exercisability of patients with ostomy, the following reports on the case of a firefighter with CRC and ostomy who regained occupational fitness through an individually tailored exercise training program. The case report was written in accordance with the Case Reporting guidelines.^[15]^

## 2 Case study

### 2.1 Patient

**Figure 1:**
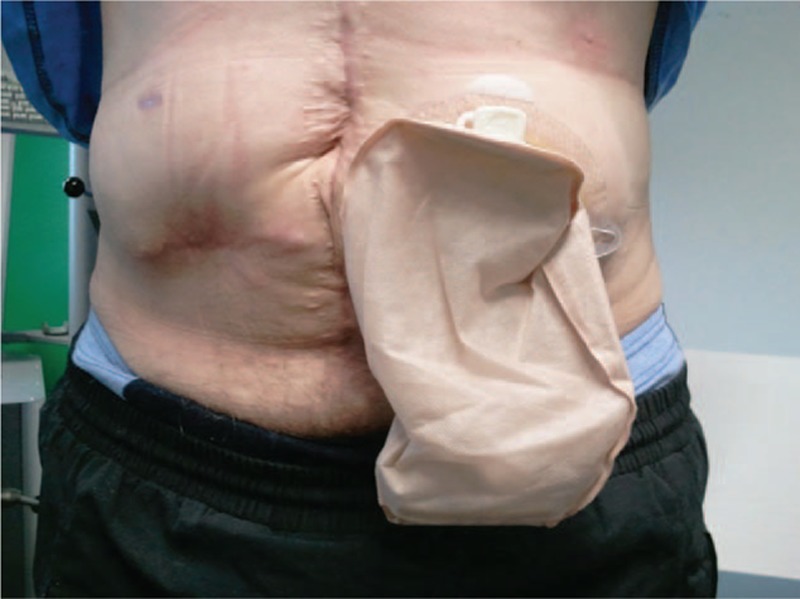
The patient's permanent descending colostomy.

A 44-year-old patient with rectal carcinoma and a permanent ostomy was submitted to the exercise expert consultation of our Comprehensive Cancer Center. Height, weight, and body mass index were 178 cm, 82 kg, and 25.9 kg/m^2^, respectively. The patient had been diagnosed with an adenocarcinoma of the rectum (uT3 cN+ cM0) 24 months prior. Following this he received neoadjuvant radiochemotherapy and surgery (ypT3 yN0 (0/16) R0) with temporary ileostomy. Due to postsurgical complications including fistulization, no adjuvant chemotherapy was administered and a 2nd temporary ileostomy was necessary after the 1st had been removed. Finally, a permanent descending colostomy at the left mid abdomen followed 12 weeks before consultation (Fig. 1).

The patient was a professional firefighter who was unable to work since diagnosis. Prior to his diagnosis, he had performed different types of aerobic (running, swimming, and rowing) and resistance training (machine based and free weight training) 5 days/week. Since diagnosis he has been completely inactive. The patient aimed to return to work with an exterior crew on a pump truck which is physically less demanding than to work with an interior firefighting crew. To prove operational fitness, he had to pass a special incremental exercise test on a cycle ergometer at the fire department's medical unit. The goal criteria for his age group were physical work capacity at 150 beats (heart rate) per minute (PWC_150_) ≥2.1 W/kg (W [watts], work rate corresponding to a heart rate of 150/min ≥2.1 W/kg body weight) and peak power output (PPO) ≥170 W. The primary aim of the exercise program and this case study was to enable the patient to reach these criteria and to pass the test successfully. The patient provides informed consent to publish this data.

### 2.2 Exercise tests

At baseline and after 2, 4, 6, and 9 months, the patient performed a set (sequence) of tests (battery) including the determination of anthropometric data (weight and height), the European Organization of Research and Treatment in Cancer questionnaire about quality of life (EORTC QLQ-C30),^[16]^ the multidimensional fatigue inventory,^[17]^ and the National Comprehensive Cancer Network distress thermometer^[18]^ as well as a standard cardiopulmonary exercise test, a strength test and a balance test which are described below. After completing the 6 months’ test, the special incremental exercise test of the fire department's medical unit was added to decide whether the patient was ready for passing his test.

The standard cardiopulmonary exercise test on a cycle ergometer started at 25 W and work rate was increased by 25 W every 3 minutes until voluntary exhaustion. A 12-lead electrocardiography, blood pressure, blood gas data (Ergostik, Geratherm Respiratory, Bad Kissingen, Germany), and capillary blood samples from the earlobe for the determination of blood lactate concentration (enzymatic-amperometric method, Biosen S-Line, EKF Diagnostic Sales, Magdeburg, Germany) were taken. PPO, maximal oxygen uptake (VO_2max_), the lactate threshold (LT), and the individual anaerobic threshold^[19]^ were determined. Intensity zones for basic endurance training were derived from the thresholds^[20]^ and described in terms of heart rates (light to moderate basic endurance training: 75%–110% LT, moderate to vigorous basic endurance training: 110% LT–97% individual anaerobic threshold).

The special incremental exercise test according to the fire department's medical unit started at 75 W and work rate was increased by 25 W/minutes. Only electrocardiography measurements were performed and PWC_150_ as well as PPO was determined.

Muscle strength was assessed on a stationary isokinetic dynamometer (IsoMed 2000 B-Series version, D&R Ferstl, Hemau, Germany). Maximal voluntary isometric contraction in knee extension, hip flexion, elbow flexion, and internal rotation of the shoulder was tested in various joint angels. Furthermore, maximal isokinetic peak torque at 60 °/second was tested in the same muscle groups.

Balance performance was assessed on a Posturomed^®^ testing system (Haider Bioswing, Pullenreuth, Germany). Anterior–posterior and medial–lateral movement of the free-hanging platform was measured in 10 seconds bipedal stands with and without perturbation. Based on the amplitude of the movements a balance score was calculated by the testing software.^[21]^

### 2.3 Training program and fitness development

Fourteen weeks after the permanent colostomy, the patient started an individually designed multimodal progressive exercise training program which is described in Table 1. All sessions started with a warm up on a cycle ergometer and ended with stretching exercises. During exercise the patient wore an ostomy belt. Except for muscle soreness no side effects or any other adverse events occurred.

**Table 1:**
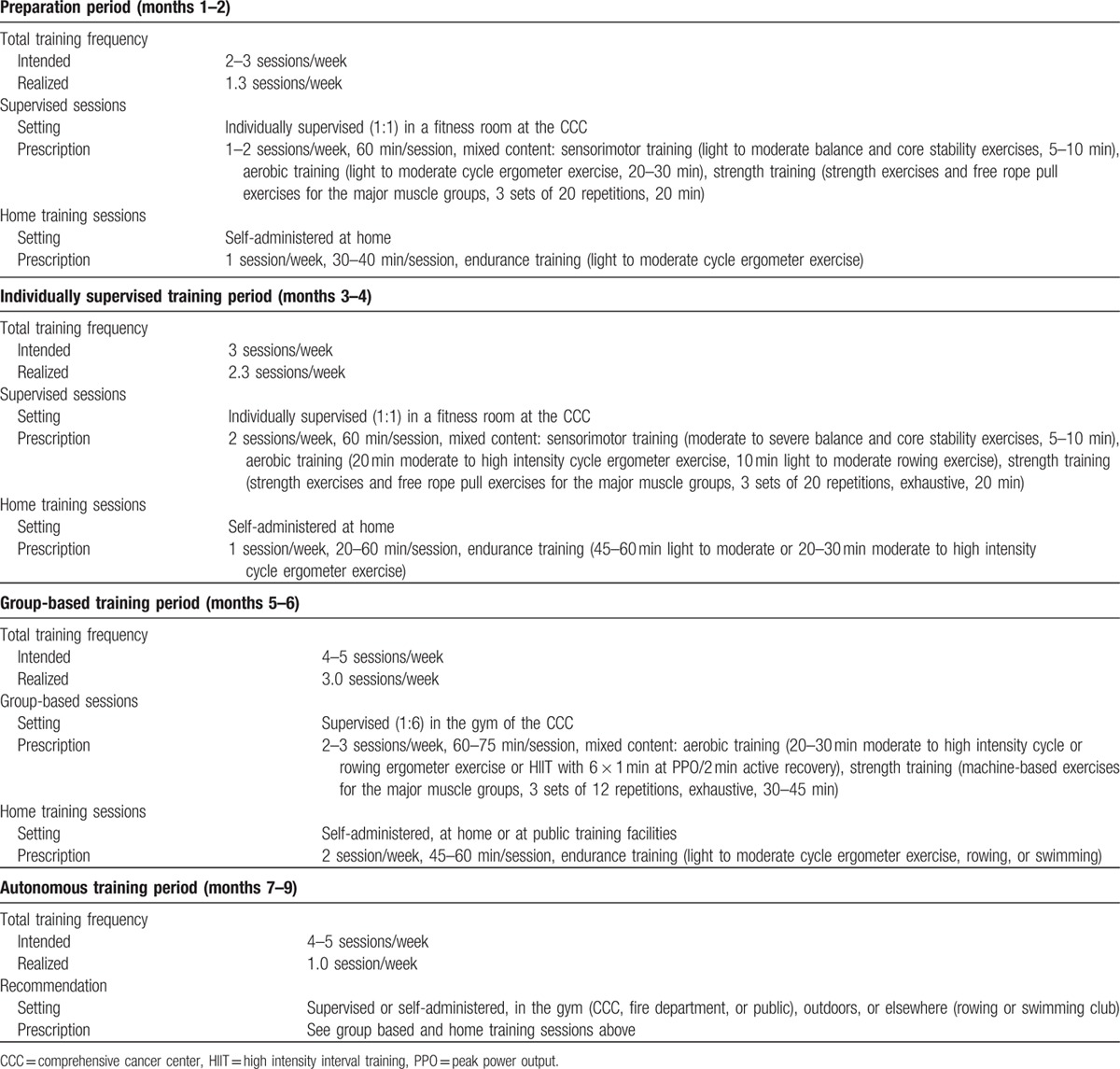
Training program.

The preparation period (baseline → month 2) focused on light to moderate sensorimotor, endurance, and strength training, taking into account the relatively fresh scar. It was planned to last 1 month, but in the beginning the patient was unable to adhere to the prescribed training frequency due to fatigue, muscle soreness, and upper respiratory tract infection, and the preparation period was therefore extended to 2 months. Furthermore, he spent considerable training time talking about his disease. Additional psychosocial counseling improved the situation. Finally, the patient managed to complete 1.3 sessions/week. The fitness development is shown in Table 2. In agreement with the low training frequency, cardiorespiratory fitness improved slightly but 3 out of 4 strength parameters improved by 13% to 30% (MPIT) and by 13% to 39% (maximal voluntary isometric contraction) in this period.

**Table 2:**
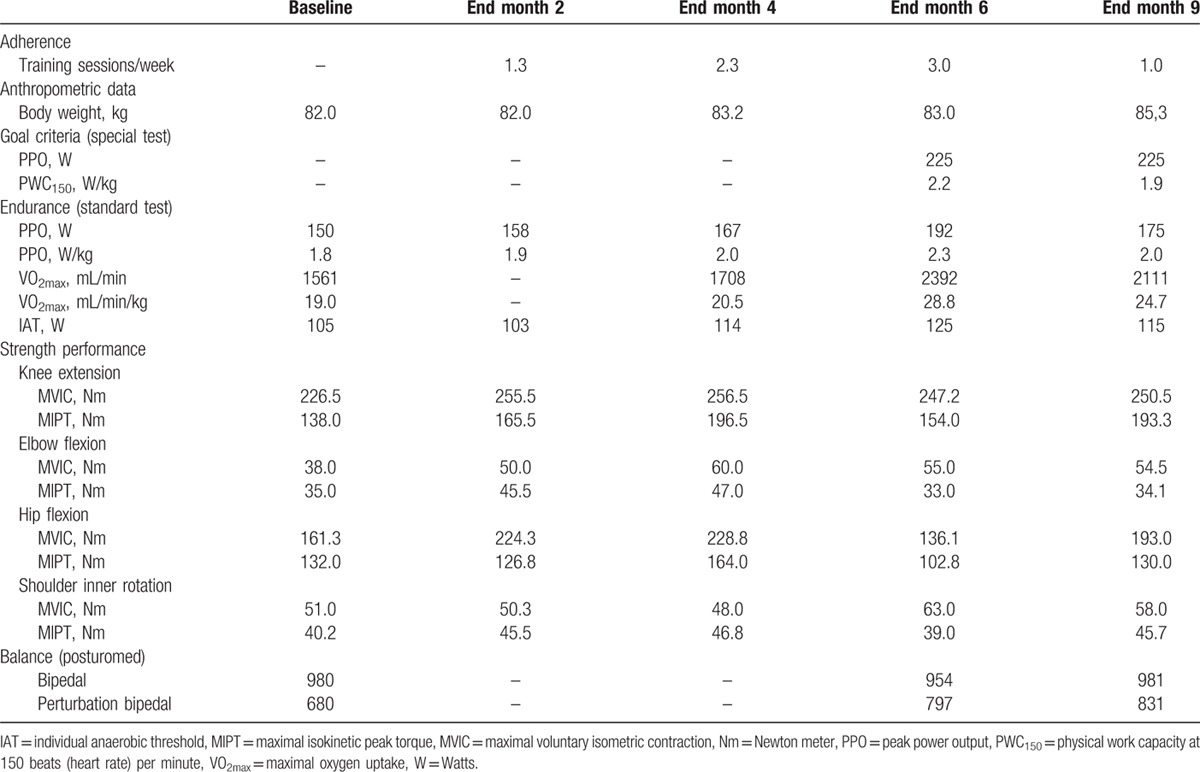
Physical performance development.

In the individually supervised training period (month 2 → month 4), training volume and intensity were increased to improve core stability, basic endurance, and strength. The patient increased his training frequency to 2.3 sessions/week, which was still lower than prescribed. As the patient experienced a slight weight gain and PWC_150_ is a body-weight related fitness criterion, he was referred to a nutritional counseling session. Fitness improved slightly (Table 2) during this period.

During the following group-based training period (month 4 → month 6), the patient exercised together with 5 to 11 other cancer survivors in our clinic and volume as well as intensity were further increased, including high intensity interval endurance training sessions. Furthermore, the variety of activities was extended to machine-based strength training, swimming, and rowing on the water. The patient further increased training frequency to 3 sessions/week, which was again less than prescribed. In this period, cardiorespiratory fitness improved considerably and the patient met the goal criteria for the operational fitness but strength performance decreased/stagnated (Table 2). The reevaluation of balance performance showed a 17% increase in the bipedal perturbation test situation. The patient scheduled his operational fitness test at the fire department's medical unit shortly after this result. He passed it and started his reintegration program at the fire department.

Thereafter, the autonomous training period (month 6 → month 9) started which aimed at maintaining fitness. The patient switched to self-organized training either in our CCC, the fire department, public sports facilities, or health clubs as he did prior to his disease. However, the reintegration program which started with working at the fire department's emergency call center led to a reduction in training frequency to only 1 session/week. Accordingly, fitness decreased again (Table 2).

### 2.4 Quality of life, fatigue, and distress

**Figure 2:**
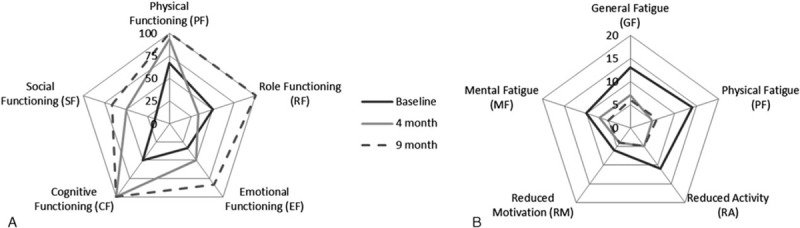
Patient reported outcomes on quality of life on EORTC functioning scale (A) and MFI subscales (B). EORTC = European Organization of Research and Treatment in Cancer, MFI = multidimensional fatigue inventory.

With regard to patient reported outcomes data at baseline, after 4 and 9 months is available. Figure 2A shows the development of the functional scales of the EORTC QLQ C30 questionnaire. Nearly all scales increased within the 1st 4 months of training except for role functioning. Global health status improved by about 33%. After 9 months, physical, role, and cognitive functioning reached maximum scores and emotional function came close to the maximum. Global health status remained stable at a score of 66.67. With regard to the EORTC symptom section, pain and diarrhea decreased by 50%, problems with insomnia and appetite loss disappeared and constipation occurred at 6 months but decreased close to nil at 9 months (data not shown). Subscales of the multidimensional fatigue inventory showed a huge fatigue reduction (between 30% in mental fatigue and 64% in physical fatigue) during the 1st 4 months of training which remained stable till month 9 (see Fig. 2B). Distress was reduced by about 50% after 9 months. At baseline, 6 physical problems (pain, fatigue, sleep, dry/itchy skin, dry/blocked nose, and sexual problems), 2 emotional problems (worries, sadness), and 1 family problem (partner) existed. After 9 months, only 2 problems in the physical section (indigestion, dry/blocked nose) remained.

## 3 Discussion

The current case study demonstrates that having a permanent colostomy does not interfere with a rectum carcinoma survivor's ability to exercise. The individualized exercise program led to improvements in physical performance, QoL, fatigue, and distress and resulted in a successful reintegration into the workplace. With regard to safety and feasibility aspects it could be proven that a sensorimotor, progressive resistance, and aerobic interval training is possible despite having a permanent colostomy since no adverse events occur.

The goal in our case study was very specific and straight forward focusing on successful reintegration into the workplace by increasing physical performance with predefined thresholds by the fire department's medical unit. Therefore, and given the fact of a case study it is hard to compare our results with the current literature. Nevertheless, our results are mainly in line with the currently available literature of exercise RCTs in CRC patients^[10,22,23]^ with regard to physical fitness development and adds evidence concerning QoL, fatigue, and distress, since Bourke et al^[24]^ were the only studies in the field showing a significant impact on fatigue. However, the authors conducted a 12-week combined intervention program of exercise and dietary advice so that the comparability to our case report is limited. Furthermore, ancillary analysis of the biggest RCT in the field supports our fatigue findings showing that participants who increased their fitness (regardless of group assignment) reduced fatigue and vice versa.^[12]^ A mixed patient group RCT with 32 advanced CRC patients (48% of the enrolled patients) showed that an 8-week home-based exercise program was also able to stabilize fatigue.^[25]^ Single arm intervention trials support the findings that exercise might reduce the severity of fatigue.^[26]^ With regard to fitness enhancement, a recently published study encourages the use of high intensity interval training as we did with the CRC survivor patient.^[27]^ However, it seems to be crucial that only 1 CRC exercise trial provides information about having an ostomy or not^[12]^ and none of the trials focused on professional rather than health-oriented exercise training.

Within our case study we had to state that although we have had a highly motivated CRC patient with the goal to return to work, reaching adequate exercise adherence levels were challenging. The patient had the need to discuss his problems concerning disease and treatment-related side-effects (e.g., ostomy) and their impact on his social environment extensively at the beginning of the study. Exercise alone did not help him to overcome these problems so that we decided to involve psychosocial services at the time. Therefore, we cannot rule out that some of the psychosocial effects shown were related to other interventions. Within the exercise treatment process it should be noted that adherence rates increased at the time when the patient was integrated into group-based exercise programs supporting the observation that being physically active is much more easier with the support of other cancer patients.^[28]^

Interestingly, with regard to exercise training response, strength increased much quicker than endurance parameters. On the one hand, radiochemotherapy and surgery might have affected organ systems more essential for endurance than for strength (i.e., cardiac and vascular system, hemoglobin concentration, and skeletal muscle oxidative capacity) and cardiorespiratory responses were therefore harder to elicit.^[2]^ On the other hand, inadequate adherence to the endurance training prescription at the beginning of the intervention could be the reason for delayed aerobic capacity response.

With regard to QoL parameters it was impressive to see that huge effects on social, role, and emotional functioning were achieved at that time when the patient returned to work. This observation underlines the need of a rehabilitative perspective in each exercise approach and relativizes the claim that QoL must be very high at the end of an exercise intervention when return to work was enabled. Interventional studies with professional rather than health-oriented exercise training might be interesting from this perspective.

## 4 Conclusion

With this case report we were able to show that having a permanent colostomy after CRC is not a barrier for returning to work with physically demanding tasks like firefighting when an individually tailored, supervised training program is administered. We were able to improve fitness, QoL, fatigue, and distress over the course of the exercise treatment without any adverse events. It has to be taken into account that this case report is on a CRC stage II patient, and it remains uncertain whether such a program can also be administered in higher stage/advance CRC patients. This should be examined in future studies. In addition, better designed, randomized-controlled exercise trials in CRC patients are warranted and future studies should precisely report on the aspect of patients having an ostomy as well as on safety and adherence issues related to health-oriented and professional exercise regimens.

## Acknowledgements

The authors thank the patient for participating in this case study and Maximilian Koeppel for supporting the exercise training sessions. We acknowledge the financial support of the Deutsche Forschungsgemeinschaft and Ruprecht-Karls-Universität Heidelberg within the funding programme Open Access Publishing.
